# The role of nanoparticle structure and morphology in the dissolution kinetics and nutrient release of nitrate-doped calcium phosphate nanofertilizers

**DOI:** 10.1038/s41598-020-69279-2

**Published:** 2020-07-24

**Authors:** Francisco J. Carmona, Gregorio Dal Sasso, Federica Bertolotti, Gloria B. Ramírez-Rodríguez, José M. Delgado-López, Jan Skov Pedersen, Norberto Masciocchi, Antonietta Guagliardi

**Affiliations:** 1 Department of Science and High Technology and To.Sca.Lab, University of Insubria, Via Valleggio 11, 22100 Como, Italy; 2 Institute of Crystallography and To.Sca.Lab, Consiglio Nazionale Delle Ricerche, Via Valleggio 11, 22100 Como, Italy; 3 Department of Inorganic Chemistry, University of Granada, Av. Fuentenueva S/N, 18071 Granada, Spain; 4 Department of Chemistry and Interdisciplinary Nanoscience Center (iNANO), Aarhus University, Gustav Wieds Vej 14, 8000 Aarhus, Denmark

**Keywords:** Bioinspired materials, Nanoparticles

## Abstract

Bio-inspired synthetic calcium phosphate (CaP) nanoparticles (NPs), mimicking the mineral component of bone and teeth, are emergent materials for sustainable applications in agriculture. These sparingly soluble salts show self-inhibiting dissolution processes in undersaturated aqueous media, the control at the molecular and nanoscale levels of which is not fully elucidated. Understanding the mechanisms of particle dissolution is highly relevant to the efficient delivery of macronutrients to the plants and crucial for developing a valuable synthesis-by-design approach. It has also implications in bone (de)mineralization processes. Herein, we shed light on the role of size, morphology and crystallinity in the dissolution behaviour of CaP NPs and on their nitrate doping for potential use as (P,N)-nanofertilizers. Spherical fully amorphous NPs and apatite-amorphous nanoplatelets (NPLs) in a core-crown arrangement are studied by combining forefront Small-Angle and Wide-Angle X-ray Total Scattering (SAXS and WAXTS) analyses. Ca^2+^ ion release rates differ for spherical NPs and NPLs demonstrating that morphology plays an active role in directing the dissolution kinetics. Amorphous NPs manifest a rapid loss of nitrates governed by surface-chemistry. NPLs show much slower release, paralleling that of Ca^2+^ ions, that supports both detectable nitrate incorporation in the apatite structure and dissolution from the core basal faces.

## Introduction

Biomineralization, the process by which living organisms generate organic/inorganic hybrids with unique properties, has long been used as an unceasing stimulus for the development of materials with new functionalities^1,2^. Among them, calcium orthophosphate (CaP) nanoparticles (NPs), the most important inorganic constituents of bone and teeth, have attracted a great deal of attention^3–6^. In vertebrates, CaP NPs appear in the form of very thin nanoplates (NPLs) of a highly structurally defective and calcium-deficient (hydroxy)apatite phase crystallizing in the hexagonal *P6*_*3*_*/m* space group^7^ (or subtle deformation thereof^8–10^), likely grown from an amorphous metastable precursor^11,12^. Synthetic CaP NPs prepared in close to physiological conditions or mimicking bone mineral structure or function (so called “biomimetic”) are remarkably biocompatible, non-toxic and biodegradable^13–15^. Additionally, they show high chemical and thermal stability, aptitude to either cation or anion doping, high adsorption capacity for organics (drugs and proteins), and pH-responsive solubility that opens the way to a controlled release of calcium and phosphate ions^6^. Owing to these remarkable properties, CaP has been used in many products for a broad range of applications, including regenerative medicine, drug-delivery, cosmetics, nano-catalysis and nuclear wastewater treatment^16,17^.

Recently, attention has been paid to CaP NPs as emerging materials able to deliver physiologically relevant macronutrients (nitrogen, phosphorus and potassium, NPK) to plants, for sustainable applications in agriculture^18–23^. Conventional fertilizers, composed of highly water-soluble salts, are severely inefficient in terms of both low plant uptake and deleterious environmental impact^24–27^. In contrast, CaP’s are sparingly soluble in water^28^ (but dissolve in acids) and are expected to undergo a slow dissolution in soils, enabling the progressive release of the active nutrients. In the form of NPs, they further exhibit high surface-to-volume ratio and easy surface functionalization. In recent pilot works, hydroxyapatite-based NPs have been proposed as slow release P-nanofertilizers (taking advantage from NPs dissolution)^18^, or co-precipitated with urea, an inexpensive and commonly used N-rich nutrient, where nitrogen release is controlled by surface chemistry^21–23,29^. These works have demonstrated that nanoapatites are ideal platforms on which diverse release mechanisms can be, even jointly, implemented. However, the role of relevant features in the solubility and dissolution rates of CaP NPs, such as their size, defects and morphology, remains unclear.

In this respect, solubility of hydroxyapatite [the most thermodynamically stable CaP, possessing the Ca_5_(PO_4_)_3_(OH) formulation in ideal crystals of geological origin^30^] is of high relevance, with important implications also in bone/teeth (de)mineralization process^6,31–35^. This is a highly complex process, controlled by many diverse factors (pH, ionic strength, Ca/P ratio, NPs size, structural defects and ionic substitutions)^28,35,36^. Progress in the understanding of this process was achieved through the evidence that apatite dissolution is accompanied by the formation of pits on the crystal surface, the density, size and spreading velocity of which influence the dissolution rate, eventually resulting into inhibition when they fall below a critical size (always at the nanoscale)^6,31–33^. These findings strongly suggest that size and defects of apatite do play an active role.

In the context of designing CaP NPs for agricultural applications, their pronounced ability of incorporating a number of exogenous ions^17^ can also be exploited to favour incorporation of macronutrients (K,N). Ionic substitution in apatite is known to proceed via Ca^2+^ replacement or by anionic exchange (oxyanions substituting OH^*–*^ or phosphates), the latter not reported, for example, in brushite (also investigated as P-fertilizer). CO_3_^2*–*^, by far the most abundant exogenous ion in bone apatite (up to 8% by weight) and one of the most critical in directing mineral crystallization/dissolution processes, can be inserted through CO_3_^2−^/PO_4_^3−^ replacement (type B) or by the rarer CO_3_^2−^/OH^−^ exchange (type A)^37,38^. Though predictably low (particularly in the presence of nearly ubiquitous CO_3_^2–^ anions), nitrogen incorporation in the form of NO_3_^–^ is of particular interest. In view of practical applications, incorporated nitrates would be readily available for plant uptake and more efficiently delivered during NPs dissolution^23^. On the fundamental side, direct evidence of NO_3_^*–*^ incorporation in highly defective apatite nanocrystals has never been given or properly discussed. Indirect EPR evidence of paramagnetic NO_3_^2*–*^ ions generated by X-ray irradiation was reported in 30 nm nanocrystals, demonstrating that replacement of phosphate ions takes place but only in trace amounts^39^. Density Functional Theory (DFT) modelling fixed that the substitution is energetically preferred in the B-type than in the A-type configuration^40^.

In the present manuscript, we focus on biomimetic CaP nanomaterials prepared with and without NO_3_^*–*^ doping. By a joint forefront analysis of small angle X-ray scattering (SAXS) and wide angle X-ray total scattering (WAXTS) data, we provide a structural, microstructural and morphological characterization at the atomic-to-nanometre detail level, and discuss the NPs dissolution, incorporation of NO_3_^*–*^ ions in the crystal lattice and release tests in relation to the structural and morphological model.

## Results and discussion

### Synthesis and characterization of N-doped biomimetic CaP nanoparticles

The biomimetic preparation of CaP NPs was carried out by aqueous precipitation performed at physiological temperature (37 °C) in the presence of carbonate ions^37^. The synthetic strategy follows green chemistry principles (mild conditions, low toxicity) and makes use of low-cost reagents, which could favour the scale-up of the process and its industrial implementation.

**Figure 1:**
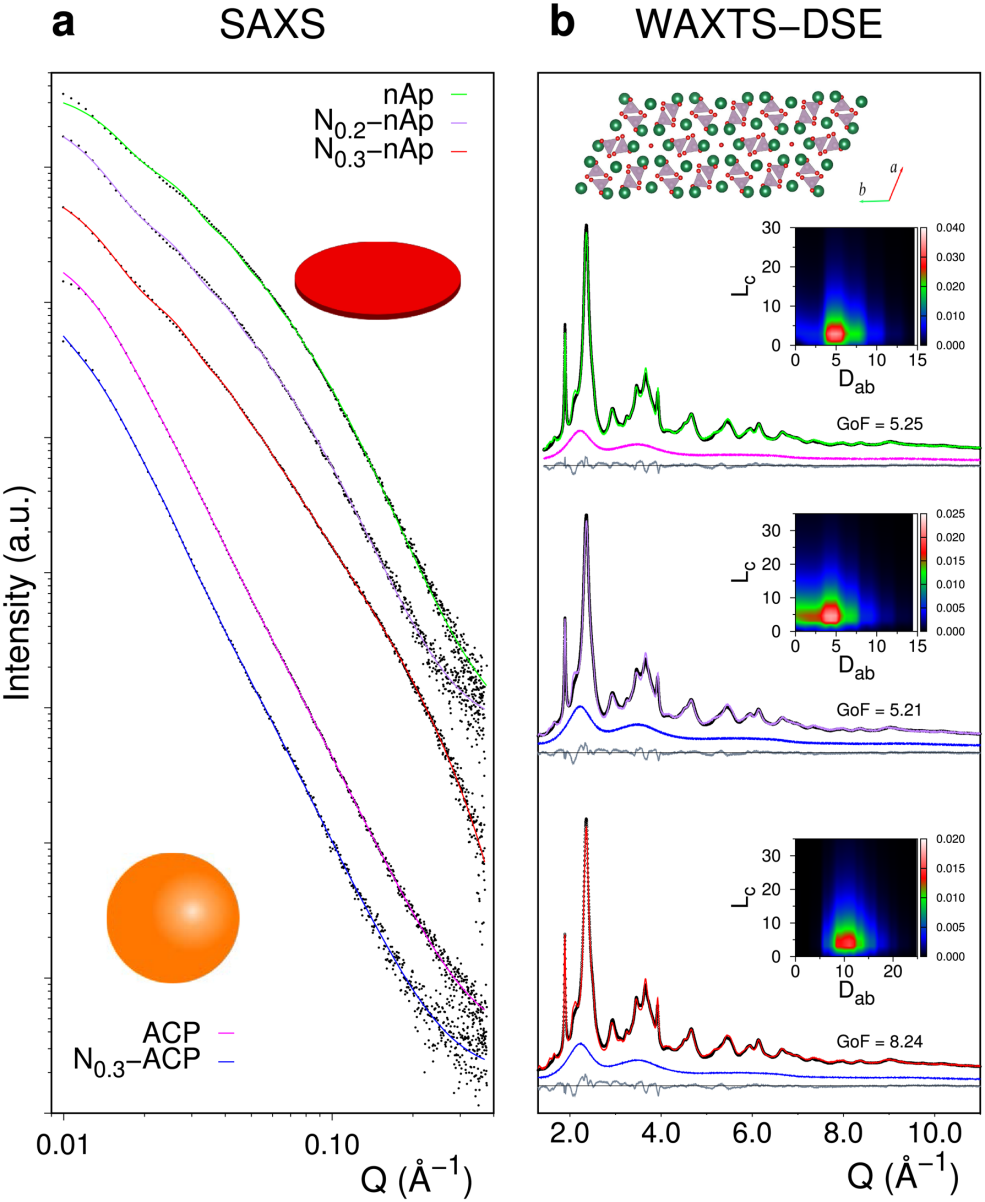
(**a**) SAXS data (log–log scale, black dots) and best models from analytical form factors of fully amorphous ACP, N_0.3_-ACP samples (pink and blue lines) and amorphous-crystalline nAp, N_0.2_-nAp, N_0.3_-nAp hybrid samples (green, violet and red lines). Fully amorphous materials are best fitted using a spherical model of NPs, the hybrids with a finite disk-shape model. Signals are shifted upwards for sake of clarity. (**b**) WAXTS data of hybrid samples (black) and DSE best fits (same colour code as in **a**) obtained by calculating the pattern from a population of atomistic models of nanoplates of apatite (top inset, view down the *c* axis); the featureless scattering pattern of the ACP and N_0.3_-ACP fully amorphous materials (pink and blue traces, respectively) is added as a model component and scaled to the experimental data. Insets: 2D map of the bivariate lognormal (number-based) size distribution (in the *L*_*c*_*,D*_*ab*_ coordinates) derived by the DSE fitting procedure for apatite NPLs, from which average sizes and size dispersions in Table 1 are obtained.

In a typical procedure, CaCl_2_ and K_2_HPO_4_ (added in an ideal hydroxyapatite ratio, i.e. with Ca/P = 1.67) were mixed in the presence of Na_2_CO_3_, resulting in the rapid precipitation of a white CaP powder. Upon maturation in solution, a purely amorphous material (5 min, labelled as ACP) or nanocrystalline apatite including an amorphous component (certified by WAXTS analysis, 24 h, labelled as nAp) was obtained (Fig. 1). We explored the feasibility of incorporating nitrate ions in the nanostructured materials in a simple one-pot synthetic approach. NO_3_^*–*^-containing CaP NPs were synthesized following the procedure described above except that Ca(NO_3_)_2,_ instead of CaCl_2_, was employed. Aiming at increasing the material functionalization, other samples were precipitated with additional amounts (0.1 M and 0.3 M) of KNO_3_ in the reaction mixture. KNO_3_ was chosen as it is an inexpensive and widely used fertilizer not altering the main synthetic conditions (Ca/P ratio, pH). The materials maturated for 24 h are labelled as N_x_-nAp, with *x* = 0.2, 0.3, 0.5 M the total NO_3_^*–*^ concentration in the pristine solutions. Synthetic conditions and compositional results are reported in Tables S1 and S2. The elemental analyses showed that the addition of 0.1 M of KNO_3_ (N_0.3_-nAp) slightly increases (by ~ 13%) the nitrate content in comparison to N_0.2_-nAp, while no further changes are observed when the KNO_3_ concentration is more than doubled (N_0.5_-nAp). The *x* = 0.3 M doping conditions were selected to prepare an entirely amorphous nitrate-doped material (5 min maturation, labelled as N_0.3_-ACP), as a reference sample for the ion release behaviour.

X-ray powder diffraction (XRPD) data of nAp and N_x_-nAp (Fig. S1) show the typical pattern of biomimetic nanoapatite and discard co-precipitation of undesired crystalline CaP phases and of unreacted salts. FTIR analysis (Fig. S2) shows several PO_4_^3−^ bands typical of apatite, additional bands associated to CO_3_^2−^ modes (indicating the partial substitution of PO_4_^3−^ groups mainly by B-type carbonate ions) and the presence of nitrate ions (ν_3_NO_3_ asymmetric stretching band at 1,384 cm^−1^)^16,17,41–44^. As diffraction data dismiss the presence of segregated phases other than amorphous calcium phosphate and apatite, nitrate ions are either adsorbed on the particle surfaces, incorporated in the crystalline/amorphous structure, or both.

In the fully amorphous ACP and N_0.3_-ACP samples, FTIR spectra (Fig. S2) show the characteristic broadening of the ν_3_ and ν_4_ vibrational modes of phosphate and carbonate ions typically found in amorphous CaP^16,42,45^. The sharp band associated to the ν_3_NO_3_ asymmetric stretching at 1,384 cm^−1^ in N_0.3_-ACP confirms the material functionalization. The relative intensities of the ν_3_NO_3_ bands in N_0.3_-ACP and N_0.3_-nAp, and the nitrate content by elemental analysis (2.12 and 0.44 w%, respectively, Table S2) indicate that longer maturation times, favouring self-healing processes, limit nitrate functionalization, in line with previous reports^23,39,40^. This result, and plant tests presented elsewhere^23^, suggest that fully amorphous NPs are best candidates for application as N-based nanofertilizers. Whether the NO_3_^*–*^ amount in the nanoapatite is retained in the amorphous or crystalline component of the 24 h-maturated CaP material is herein investigated (see below).

### Joint SAXS and WAXTS analyses

An amorphous component was found to co-exist with the crystalline apatite phase in nAp and N_x_-nAp materials by WAXTS analysis (Fig. 1). Whether this amorphous component occurs as a surface layer of the apatite core (in a core–shell arrangement), as reported for bone mineral^46–48^, or as distinct particles is not accessible by WAXTS. Taking inspiration from recent studies, a combination of SAXS and WAXTS analyses was adopted (Fig. 1)^49^. SAXS data are sensitive to electron density contrast at the nanoscale level but are intrinsically blind to atomic scale features, therefore enabling the investigation of the NPs morphology regardless of their crystalline or amorphous nature.

SAXS analysis based on analytical form factors (related to the average size and shape of NPs), and a structure factor (associated to concentration effects)^50^ revealed a distinctive platy morphology of NPs for the nAp and N_x_-nAp samples. Information on the average thickness (*T*) was obtained through a finite disk-shape model having much larger and monodisperse diameter (Fig. 1a and Table S3). Discs exhibiting nearly equal thickness (*T* ~ 2.3 nm, number-based averages determined according to a Zimm-Schulz distribution^51^) and rather polydisperse (*σ/T* ~ 50%) best matched the nAp and N_0.2_-nAp data. A bimodal model including thicker (*T* = 4.5 nm) and thinner (*T* = 1.5 nm) platelets in a 2:1 volume proportion (both exhibiting lower *T* dispersion, *σ/T* ~ 30%), best described the N_0.3_-nAp data. This finding and the parallel WAXTS analysis (see below) jointly highlight the composite nature of platelets in nAp and N_x_-nAp samples, suggesting the intimate coexistence of the crystalline and amorphous components. SAXS analysis of the pure amorphous samples (ACP and N_0.3_-ACP) indicated the occurrence of a single (highly disperse) population of spherical NPs, undetected in the nanoapatite materials (Fig. 1a). These evidences are further corroborated by TEM imaging (Fig. S3), where the co-existence of the two distinct morphologies was never observed.

SAXS data were also treated by the Debye Scattering Equation (DSE)^52^ method that relies on an atomistic model of discrete apatite nanocrystals developed to perform WAXTS data analysis^5^. The DSE-based SAXS modelling benefits from the morphological description of NPs with the noticeable advantage of enabling the direct comparison of size parameters from the small to the wide-angle regions^53,54^. Using the apatite crystal structure^7^, populations of nanocrystals having platy morphology (NPLs) were built, with thickness (*T*), width (*W*) and length (*L*) aligned to the 2***a*** + ***b*** *(*or a*), ***b*** and ***c*** crystallographic axes, respectively, fixed *T/W* aspect ratio in *ab* and two independent growth directions (in the *ab* plane and normal to it, that is along *c*). Details are given in Supplementary Methods and results summarized in Table 1, Table S4 and Fig. S4. Notably, the thickness of the NPLs well agrees with those of discs derived by conventional SAXS analysis (Table S3).

**Table 1:** Number-based average thickness (*T*), width (*W*) and length (*L*) and relative dispersions (*σ*_*T*_*/T* = *σ*_*W*_*/W*, *σ*_*L*_*/L*) of entire NPLs (from SAXS-DSE) and apatite (Ap) NPLs (from WAXTS-DSE); the average volume (*V*) of nanoparticles and nanocrystals is obtained as the weighted sum of each particle/crystal volume, according to the lognormal distribution.

SAXS	*T* (nm)	*W* (nm)	*σ*_*T*_*/T* = *σ*_*W*_*/W* (%)	*L* (nm)	*σ*_*L*_*/L* (%)	*V* (nm^3^)	*T*_*SAXS*_–*T*_*Ap*_ (nm)	*W*_*SAXS*_–*W*_*Ap*_ (nm)	*L*_*SAXS*_–*L*_*Ap*_ (nm)
nAp	2.09	16.90	50.14	9.80	90.24	436	0.08	2.97	0.99
N_0.2_-nAp	2.00	16.14	65.06	11.73	83.70	538	0.05	4.85	1.13
N_0.3_-nAp	4.49	36.31	37.48	30.76	11.07	5,698	0.16	11.33	19.64
	1.37	11.08	37.78	40.03	4.13	692	0.02	3.30	28.91

WAXTS	*T* (nm)	*W* (nm)	*σ*_*T*_*/T* = *σ*_*W*_*/W* (%)	*L* (nm)	*σ*_*L*_*/L* (%)	*V* (nm^3^)	*ACP* (wt%)	*Ap* (wt%)	*(V*_*SAXS*_*-V*_*Ap*_*)/V*_*SAXS*_ (%)
nAp	2.01	13.93	47.56	8.81	100.09	308	31.71	69.29	29.31
N_0.2_-nAp	1.95	11.29	56.03	10.60	84.10	309	39.31	61.69	42.55
N_0.3_-nAp	4.33	24.98	30.62	11.12	88.94	1,310	34.70	39.18	–
	1.35	7.78	34.26	11.12	88.94	132		26.12	

Size differences of SAXS and WAXTS values account for the core-crown-like model illustrated in the main text. ACP and Ap quantifications, from WAXTS analysis, are given as weight percentages (wt%). The difference between *V*_*SAXS*_ (measuring the entire NPL volume) and *V*_*Ap*_ (referring to the crystalline fraction only) nearly matches the ACP wt%. Major aggregation effects of the N_0.3_-nAp sample (influencing the SAXS average length) make the volume comparison unreliable.

**Figure 2:**
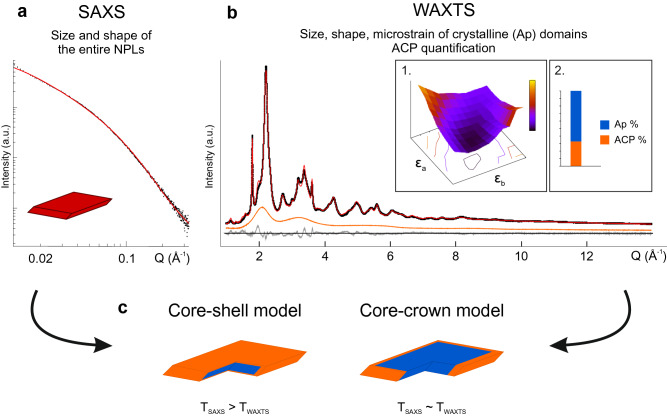
Schematic of combined SAXS- and WAXTS-DSE analysis leading to a comprehensive quantitative modelling of CaP nanocomposites: (**a**) size and morphology of hybrid amorphous-crystalline NPLs by SAXS analysis; (**b**) WAXTS modelling of apatite NPLs (encoding size, morphology, lattice strain within a unified atomistic model) and amorphous quantification; (**c**) validating the core-crown vs the core–shell crystalline-amorphous arrangement.

Synchrotron WAXTS data of nAp, N_0.2_-nAp and N_0.3_-nAp (Fig. 1b) exhibit the typical features of nano-sized and structurally disordered biomimetic apatite, with broad Bragg peaks and a high amount of diffuse scattering. In contrast to Rietveld-based analysis modelling only Bragg scattering, a complex DSE-based model, allowing Bragg and diffuse scattering to be treated on an equal basis, simultaneously conveys structure, defects, size and shape features^55–57,59^. Subtle structural deformations lowering the hexagonal apatite symmetry^8,9,10^ are hindered by finite-size and crystal defectivity and, therefore, neglected in the atomistic models. Our analysis disclosed a clear platy morphology for the apatite component of nAp and N_x_-nAp samples. The NPLs model used in SAXS analysis was applied and *T*, *W* and *L* sizes were optimized against the WAXTS data during the DSE fitting procedure^5,58,59^. Additional peak broadening attributed to lattice strain was treated by modifying the NPLs at the atomistic level^60^. An anisotropic lattice strain model was considered, according to a second-order rank strain tensor matching the morphological symmetry (*ε*_*a*_ = *Δa/a* ≠ *ε*_*b*_ = *Δb/b ≠ ε*_*c*_ = *Δc/c*), jointly to the exploration of the hypersurface of strain parameters^59^ (Fig. 2b; details in Supplementary Methods). The best strain values (Table S4) show systematically lower ε_c_ values (0.3%-0.4%) in all samples, indicating a limited *c* axis distortion, in line with previous reports^61,62^. This strain is mainly ascribed to CO_3_^2−^/PO_4_^3−^ replacements, detected in similar amount in all samples (Table S2). Incorporation of NO_3_^*–*^does not dramatically concur with increasing the overall apatite structural disorder. Comparable strain values were reported in synthetic (heavily carbonated) and natural (human enamel) samples^63,64^. The contribution to scattering of the amorphous component, likely forming a surface layer around the apatite core^46–48^, was managed in the DSE analysis by scaling the synchrotron X-ray pattern of a fully amorphous material to the WAXTS data (jointly to the calculated signal of apatite), as shown in Fig. 1b. By scaling this curve, the amount of ACP fraction was obtained, upon normalization to electron units. Table 1 quotes the results of the WAXTS-DSE analysis in terms of average sizes of the apatite core, their relative dispersions (according to a bi-variate lognormal distribution shown as inset of Fig. 1b) and the ACP wt% for the best fits.

A comprehensive picture of the amorphous-apatite hybrid in nAp and N_x_-nAp samples was derived by combining size results from SAXS and WAXTS analysis, as schematically shown in Fig. 2. Current models of bone mineral and synthetic bio-inspired analogues suggest a core–shell arrangement with the amorphous forming about 1–2 nm thick layer on top of the apatite basal faces^47,48^. Based on the thickness matching of our SAXS/WAXTS analysis (Fig. 2c), two simplified models can eventually be considered: a core-crown (*T*_*SAXS*_ ≈ *T*_*WAXTS*_) and a core–shell (*T*_*SAXS*_ > *T*_*WAXTS*_) model, allocating the amorphous component mainly along the width and length edges of NPLs or more homogeneously around the crystalline core, respectively. Vanishing or very small discrepancies between *T*_*SAXS*_ and *T*_*WAXTS*_ were found (Table 1), with major deviations residing in the *W* and *L* parameters. Accordingly, although an ultrathin amorphous (or highly disordered) layer located on the extended facets of apatite NPLs cannot be ruled out, this finding suggests that the core-crown model is most appropriate for our samples. A similar finding has been recently reported in bone apatite^59^.

Notably, by reasonably assuming a negligible electron density difference between ACP and apatite, the total volume of the NPLs estimated by SAXS-DSE, compared to that found for the crystalline part, should account for the amorphous phase in a similar proportion. These volume differences, provided in Table 1, are in fair agreement with the above (independently determined) quantification (except for N_0.3_-nAp, owing to major aggregation effects), giving additional consistency to our combined SAXS/WAXTS models.

### Lattice variations by NO_3_^*–*^-doping

**Figure 3:**
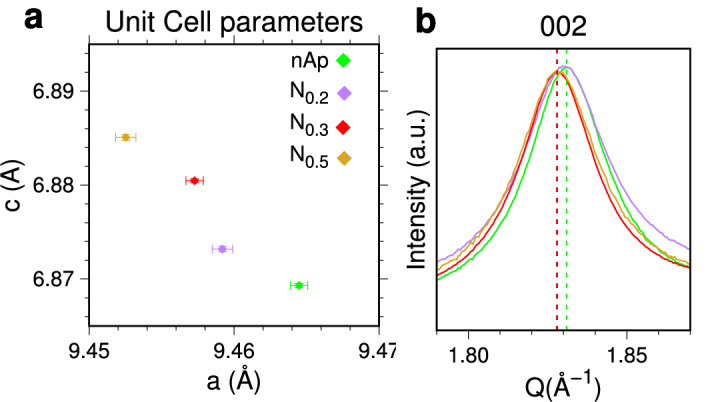
(**a**) Progressive *c* axis expansion and concomitant *a* axis contraction of apatite unit cell in NO_3_-doped N_x-_nAp samples in comparison to (NO_3_-free) nAp, upon joint carbonate and nitrate doping. Error bars as provided by Rietveld least-squares refinement; (**b**) 002 peak shifting towards lower *Q* values in N_x-_nAp *vs* nAp.

Relative changes of the *a* and *c* lattice parameters are often used as indicative of different types/extent of substitution in the apatite crystal structure^5,65^, e.g. CO_3_^2*–*^ replacing PO_4_^3*–*^ or OH^−^ in carbonated samples^19,41,61,66^, or other ions (Na^+^, K^+^ and divalent metals) present in cationic sites, though inserted in much smaller amount^67,68^. Concerning NO_3_^*–*^ ions, their incorporation in apatite has been demonstrated by previous experimental and computational studies^39,40^. However, specific effects on the crystal lattice have not been reported, likely due to experimental difficulties in detecting tiny shifts of broad Bragg peaks by conventional XRPD. Here, doping-induced changes went easily detected using synchrotron WAXTS data with high-angular resolution. Upon increasing the concentration of NO_3_^*–*^ precursors, the progressive *c*-axis expansion coupled to the concomitant *a*-axis contraction is found (Fig. 3a). A similar behaviour is reported as primary effect of B-type CO_3_^2−^/PO_4_^3−^ substitution in bone mineral and biomimetic apatite^5,37,41,61^. Given that carbonate ions are present in much larger amount than nitrate in the N_x_-nAp samples (Table S2), that the measured effects are solely due to carbonate ions cannot be ruled out. However, considering that: (1) nearly comparable CO_3_^2*–*^ levels are quantified by elemental analysis (Table S2) mainly in B-type substitution (Fig. S2), in both nitrate-free and nitrate-doped samples; (2) similar quantification of amorphous and crystalline components is estimated and (3) crystal domains have similar sizes and shapes (Table 1) in the different samples, the measured unit cell variations may reasonably be attributed to simultaneous NO_3_^*–*^/CO_3_^2*–*^ incorporation in the apatite lattice. The joint effect of this multiple replacement is clearly perceptible on the *002* Bragg peak of N_x_-nAp (Fig. 3b), shifted to lower angles in comparison to the material incorporating carbonate only.

Thus, NO_3_^*–*^ ions, thanks to comparable stereochemical features (thermochemical radii: *R*_*nitrate*_ = 179 vs* R*_*carbonate*_ = 178 pm^69^) enter the crystal structure through a mechanism similar to that observed for B-type carbonate ion, as predicted by DFT^40^. However, by diffraction methods, nitrate incorporation in the crystal lattice cannot be quantified and, owing to the amorphous/crystalline nature of nanoparticles, the relative partitioning of nitrate between the two components cannot be assessed. Notably, in the competition between the two different ionic substitutions, CO_3_^2*–*^ is significantly favoured over NO_3_^*–*^ when a biomimetic synthesis is adopted^39^, where carbonate is deliberately added to control the size and the solubility of apatite NPs.

### Chemical stability and dissolution kinetics

The fully amorphous N_0.3_-ACP NPs and the nAp and N_0.3_-nAp hybrid NPLs were selected to investigate sample stability and their dissolution properties. While it is well-established that bulk apatite is insensitive to exposure to water at neutral and basic pH values, ACP has been repeatedly reported to be chemically unstable in these conditions, its transformation into hydroxyapatite being thermodynamically favoured^6^. However, different factors (including, among others, pH, temperature, ionic strength or organic additives) affect ACP stability and particularly its transformation rate^70,71^.

**Figure 4:**
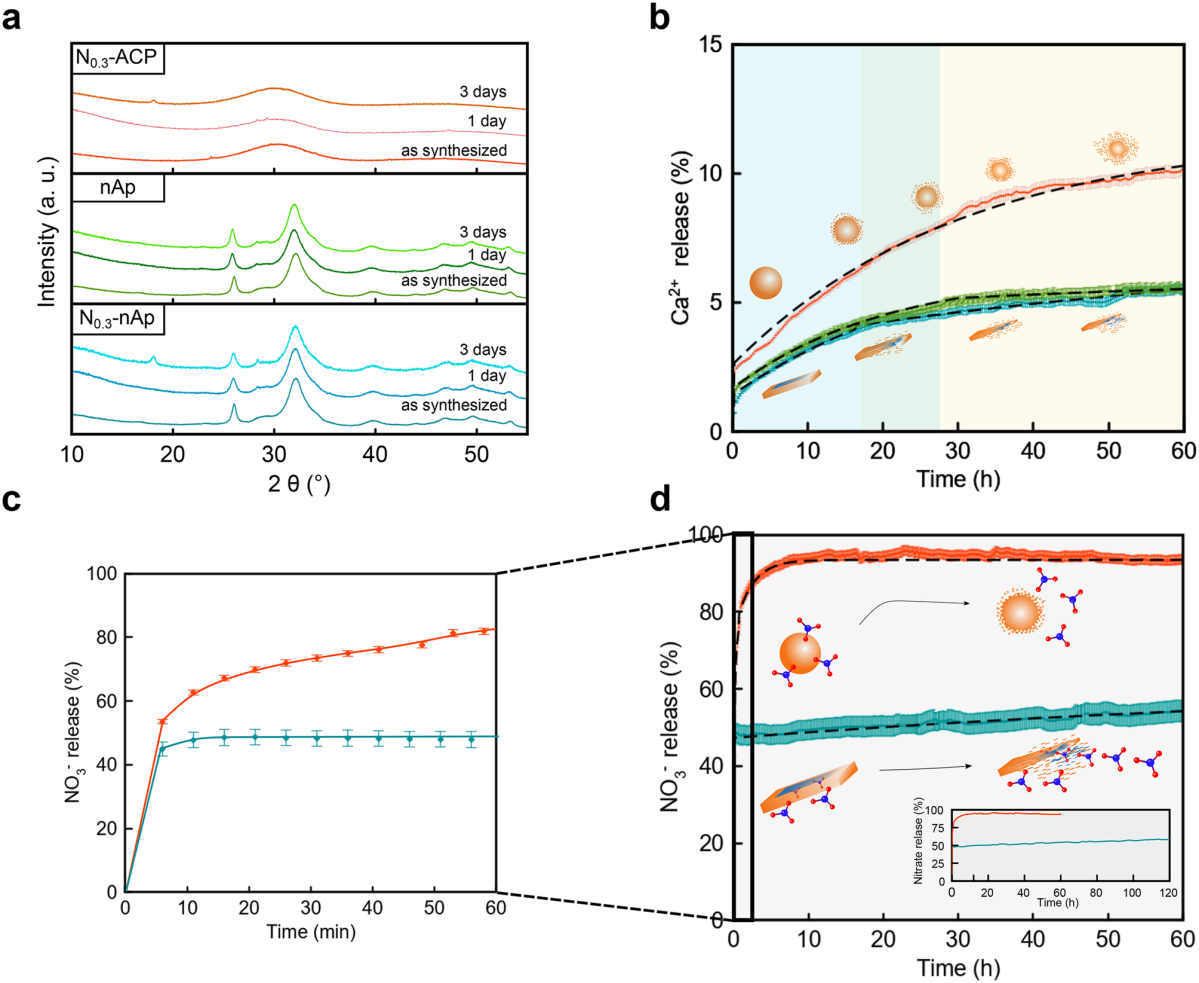
Study of the structural stability of, and ion release from, CaP NPs suspended in water at room temperature. (**a**) XRPD diffractograms of as synthesized N_0.3_-ACP, nAp and N_0.3_-nAp, after 1 and 3 days of suspension; (**b**) kinetics of dissolution of N_0.3_-ACP (orange curve), nAp (green curve) and N_0.3_-nAP (blue curve) NPs by quantification of Ca^2+^ released *versus* time. The quantification is given as % of the total amount of calcium in each sample; (**c**,**d**) kinetics of nitrate ion release of N_0.3_-ACP (orange curve) and N_0.3_-nAp (blue curve). The NO_3_^−^ is given as % of the total amount of nitrate in each sample; the burst release observed in the first minutes (**c**) is associated to weakly adsorbed NO_3_^−^ groups. Error bars in (**b**) and (**d**) correspond to 3% relative error for calcium and less than 5% relative error for nitrate.

The structural evolution of the selected materials was monitored by suspending the powders in water, at 22 °C under stirring, and characterizing by XRPD (Fig. 4a) and Fourier-transform infrared spectroscopy (FTIR, Fig. S5) the dried solid fractions recovered via centrifugation after 1 and 3 days. Figure 4a shows that N_0.3_-ACP keeps being purely amorphous, certifying that the ACP-to-apatite transformation does not take place. Similarly, XRPD and FTIR traces of the nAp and N_0.3_-nAp NPLs are virtually unchanged, with the notable exception, in the 3-days nitrate-doped samples, of a very minor precipitation of an unknown material, not attributed to any calcium phosphate or nitrate phase.

The release of Ca^2+^ and, separately, of NO_3_^−^ ions were potentiometrically determined using ion-selective electrodes. For calcium, aqueous suspensions of the nanomaterials were stirred at room temperature and the evolution of [Ca^2+^] was monitored up to 60 h, taking care that changes in concentrations were not dependent on partial water evaporation. As CaP degradation simultaneously releases both Ca^2+^ and PO_4_^3−^ ions, quantifying the amount of dissolved Ca^2+^ makes the estimation of the PO_4_^3−^ release kinetics possible. Therefore, the in vitro behaviour of nanoparticles as potential P-nanofertilizers can also be assessed.

The Ca^2+^ release profiles (given in Fig. 4b as % of the total amount of calcium in each sample) indicate that all the selected nanomaterials are partially soluble in water (at the starting operational pH ~ 6.0, progressively increasing to pH ~ 7.0), freeing up to 10.1 (N_0.3_-ACP), 5.5 (nAp) and 5.6% (N_0.3_-nAp) of the entire content of calcium after 60 h. Figure 4b also reveals that dissolution rates decrease but never vanish; thus, further dissolution is expected for longer exposure times. The concentrations of Ca^2+^ after 60 h (≤ 22 mg l^−1^) are still well below the saturation value (74 mg l^−1^) estimated for carbonated apatite (see Supplementary Methods)^72,73^.

The dissolution behaviour is likely influenced by a number of factors, such as the high content of carbonate ions (inducing evident lattice strain)^65^, the occurrence of the more soluble amorphous phase, the structural disorder (multiple doping, Ca^2+^ deficiency) and the operational pH provided by our slightly acidic deionized water^29,72,74^. Table 2 synoptically collects some of the most relevant factors driving dissolution in the selected samples: the specific surface areas (SSA_SAXS_) were calculated using size dispersion provided by SAXS-DSE analysis (Table 1); separate values for the basal and lateral area of NPLs are also calculated. These total SSA values, of about 350 m^2^ g^−1^ for NPLs, are overestimated in comparison to those experimentally determined in bone and synthetic apatite (100–200 m^2^ g^−1^)^12^ since, by neglecting aggregation, all the NP surfaces contribute to the calculation. As further reference, experimentally determined SSA of baboon bone sample (110.83 m^2^ g^−1^) and synthetic carbonated apatite (56.55 m^2^ g^−1^) are given^33^.

**Table 2:** Nitrate and carbonate content, Ca/P ratio, specific surface area (SSA_SAXS_) and kinetic constants (*k*_*dis*_ and *k*_*rel*_) of selected samples, relevant to NPs dissolution and ion-release. SSA_SAXS_ are calculated from sizes of NPs using the entire population, each NP being weighed by its distribution number fraction.

Sample	NO_3_^−^ (wt%)^a^	CO_3_^2−^ (wt%)^a^	Ca/P ^b^ ratio	SSA_SAXS_, m^2^ g^−1^			*k*_*dis*_, Ca^2+^ release (h^−1^)	*k*_*rel*_, NO_3_^−^ release (h^−1^)
				Basal	Lateral	Total		
N_0.3_-ACP	2.12	7.44	1.62	–	–	83	0.031	Burst, 0.450
nAp	0.00	7.40	1.65	247	103	350	0.048, 0.013	–
N_0.3_-nAp	0.44	7.34	1.62	291	62	353	0.035, 0.011	Burst, 0.010

For composite nanoplates basal and lateral values are also provided.

^a^From elemental analysis.

^b^Calculated from ICP-OES.

The cumulative Ca^2+^ release curves shown in Fig. 4b clearly indicate heavy deviations from classical transport- or surface-controlled kinetic models, which would imply concentration profiles of the released ions following a √*t* or *t* dependence, respectively^75^. Instead, a relatively rapid decrease of dissolution rates, even in undersaturated conditions, is observed in our samples, indicating a self-inhibiting mechanism similarly to what found in fluoroapatites, bone and synthetic apatite crystals^31–33^. In the present work, we describe the significantly bent release profiles with phenomenological pseudo-first order kinetic models (*i.e.,* [Ca^2+^] ∝ 1 − e^*−kt*^), and compare the derived kinetic constants, *k*_*dis*_, taken as numerical indicators of the progressively levelling of the released ion amount. Using this approach, significant differences between the fully amorphous and amorphous-crystalline hybrid materials can be appreciated in terms of their dissolution kinetics (see Table 2).

As expected, N_0.3_-ACP exhibits a faster dissolution rate than the hybrid NPLs^76^; its Ca^2+^-delivery profile is fitted by pseudo-first order kinetics with constant *k*_*dis*_ = 0.031 h^−1^. In contrast, nAp and N_0.3_-nAp NPLs show an overall lower level of dissolution, both exhibiting two different kinetic regimes. Initially, the *k* constants (*k*_*dis-1*_ = 0.048 h^−1^, *t* < 28 h for nAp; *k*_*dis-1*_ = 0.034 h^−1^, t < 16 h, for N_0.3_-nAp) are somewhat comparable to that of the fully amorphous N_0.3_-ACP sample, whereas at prolonged time the dissolution process slows down, with more than halved *k* values (*k*_*dis-2*_ = 0.013 h^−1^; *k*_*dis-2*_ = 0.011 h^−1^). The parallel behaviour of undoped and nitrate-doped samples suggests that doping does not influence significantly the dissolution rates of NPLs.

The cumulative amount of Ca^2+^ ions released by the NPLs might be reasonably attributed to their amorphous fraction only, owing to its higher solubility. However, by normalizing the relative percentage of Ca^2+^ delivered by N_0.3_-ACP at 60 h (10.1%) to the fractions quoted in Table 1, smaller amounts (3.2% *vs*. 5.5% in nAp and 3.5% *vs*. 5.6% in N_0.3_-nAp) of Ca^2+^ ions should have been measured. This finding suggests that the dissolution proceeds faster in the NPLs than in spherical NPs. Such a difference might be explained by the larger total (or even lateral) SSA_SAXS_ of the former (see Table 2). However, considering that the basal faces of NPLs are much wider than the lateral ones and that they expose to a large extent apatitic cores (according to the core-crown model), an alternative (and more intriguing) hypothesis can be formulated: after initial faster dissolution of the peripheral amorphous component, calcium ion release mainly proceeds through dissolution of basal faces encompassing also the highly defective crystalline core. Notably, this hypothesis would not disagree with models predicting dissolution reduction, or even termination, for small nanocrystal sizes^32,33^. Here, the extremely reduced NPLs thickness (*T* < 4 nm) could inhibit further dissolution from the crown, leaving basal faces prone to slower ion release. Presently, neither of the two proposed hypotheses conflict with existing models of dissolution of CaP in undersaturated conditions, which were mainly developed for crystalline, rather than for amorphous or hybrid materials. In this regard, monitoring dissolution by in situ SAXS/WAXTS and applying quantitative models as those here proposed would help to clarify the process.

A parallel study of NO_3_^*–*^ ions release was conducted on the amorphous N_0.3_-ACP NPs and on the hybrid N_0.3_-nAp NPLs, intended for potential applications in agriculture and to further investigate the effect of nitrate-doping in nanoapatite. Figure 4c,d show the cumulative release profiles displaying very different traces for N_0.3_-ACP and N_0.3_-nAp, expressed as the amount of released NO_3_^*–*^ ions over their total amount within the sample (see Table 2).

In both cases, an initial burst releases about 50% of the total nitrate and is attributed to ions loosely trapped into the inter-grain spaces. Afterwards, a more controlled release process (*k*_*rel*_ = 0.450 h^−1^) is observed in N_0.3_-ACP that, however, exhibits a release rate almost 15 times faster than nanoparticle dissolution (*k*_*dis*_ = 0.031 h^−1^). Indeed, almost the entire content of nitrate (94.2%) is released within the first 10 h, with a plateau being measured for longer times (Fig. 4d). This finding points to the fact that the larger amount of nitrate found in N_0.3_-ACP, compared to that stored in N_0.3_-nAp, is weakly bound to the NPs surface and that the nitrate-release process must be largely governed by the desorption of the dopant from the particle surface, much more important than that generated by particle degradation.

In contrast, after the initial burst event, the rate constant of the N_0.3_-nAp nanocomposite lowers to *k*_*rel*_ = 0.010 h^−1^, comparable to that of Ca^2+^ release observed in the second regime (*k*_*dis-2*_ = 0.011 h^−1^), suggesting a mechanism of nitrate release strongly connected to NPLs degradation and a much stronger chemical interaction with neighbouring cations.

These observations endorse additional considerations on nitrate doping in relation to the two alternative nanocomposite dissolution models previously discussed. According to the model governed by the dissolution of the amorphous portion, its continuous and slow nitrate release would require a strong(er) NO_3_^*–*^ binding, difficult to explain within an amorphous structure. At variance, the alternative model where late dissolution, observed by Ca^2+^ ion release, is governed by the basal faces of the NPLs would better explain the highly decelerated release profile by the effective incorporation of nitrate ions in the crystal lattice, as also witnessed by variations of lattice parameters beyond carbonation effects.

## Conclusion

Biomimetic calcium phosphates have been precipitated as purely amorphous spherical NPs or thin amorphous-crystalline NPLs, depending on maturation time. A complex atomic-to-nanometer scale modelling, combining X-ray scattering experiments in the small and wide-angle regions, enabled us to extract the thickness, width and length of the hybrid NPLs and their crystalline part and to infer the mutual relationship of the two components, interconnected in a core-crown-like structure. Ca^2+^ ions release tests from purely amorphous NPs, nitrate-free and nitrate-doped NPLs demonstrate that all materials show a self-inhibiting dissolution (in undersaturated aqueous solution), where the dissolution rate is driven by the nature and morphology of NPs. Additionally, small amounts of nitrate ions can be incorporated in the apatite crystal structure by an ionic substitution mechanism resembling that of carbonate ions, which introduces detectable variations of the lattice parameters beyond carbonation. A gradual release of nitrate, measured in water, follows the parallel dissolution of nanocomposites over days. In contrast, in purely amorphous materials, nitrate ions (weakly adsorbed on the surface) are mostly released within the first ten hours.

These results, addressed to the use of CaP powders as nanofertilizers, may have implications also on the bio- and de-mineralization mechanisms of bone and teeth.

## Methods

### Materials

All materials were commercially available and used without further purification. Calcium chloride dihydrate (CaCl_2_·2H_2_O, BioXtra, ≥ 99.0% pure), calcium nitrate tetrahydrate (Ca(NO_3_)_2_·4H_2_O, BioXtra, ≥ 99.0% pure), anhydrous dipotassium hydrogen phosphate (K_2_HPO_4_, ACS reagent, ≥ 98.0), sodium carbonate (Na_2_CO_3_, BioXtra, ≥ 99.0% pure), potassium nitrate (KNO_3_, BioReagent) were supplied by Sigma-Aldrich. In all syntheses, type 1 water (with conductivity < 0.06 μS/cm) was used.

### Synthesis of calcium phosphate nanoparticles

In a typical procedure, two aqueous solutions (1:1 v/v, 100 mL total) of (1) 0.2 M CaCl_2_·2H_2_O and (2) 0.1 M Na_2_CO_3_ + 0.12 M K_2_HPO_4_ were mixed at room temperature. The mixture was introduced in a glass bottle sealed with a screw cap (Duran) and heated at 37 °C for 5 min (ACP) or 24 h (nAp). At each time, resulting solids were collected by centrifugation (10 min, 5,000 rpm), washed with water (1 × 100 mL, 1 × 50 mL), freeze-dried (LyoQuest, Telstar, Spain) and stored at room temperature in sealed vials. The addition of carbonate to the reaction mixture maintains the proper pH value (~ 7.5), leads (upon 24 h maturation) to precipitation of nanosized apatite resembling the mineral component of bone and avoids the undesired precipitation of octacalcium phosphate, Ca_8_H_2_(PO_4_)_6_.5H_2_O, or brushite, CaHPO_4_·2H_2_O, obtained in more acidic solutions^6,77^. Possible carbonate absorption from the ambient is expected to be much lower than the concentration of carbonate added to the solution (13.2 µM vs. 0.05 M) and therefore was neglected. Experimental evidence on similar syntheses also demonstrates that incorporation of carbonate from ambient does not exceed 1 wt%^5^.

### Synthesis of nitrate-doped calcium phosphate nanoparticles

The nitrate doping of calcium phosphate nanoparticles was carried out by a one-pot process, following a similar procedure than in CaP preparation. Firstly, a synthetic screening was carried out to evaluate the ability of resulting CaP nanoparticles to incorporate nitrate groups. With this aim, two aqueous solutions (1:1 v/v, 100 mL total) of (1) 0.2 M Ca(NO_3_)_2_·4H_2_O and (2) 0.1 M Na_2_CO_3_ + 0.12 M K_2_HPO_4_ + *a* KNO_3_ (*a* = 0 M, 0.2 M or 0.6 M, for N_0.2_-nAp, N_0.3_-nAp and N_0.5_-nAp, respectively) were mixed at room temperature and heated at 37 °C during 24 h. The resulting solids were collected and stored as described above. The optimal conditions were selected as *a* = 0.2 M (whole nitrate concentration in the reaction (x), x = 0.3 M). Therefore, a reaction with *a* = 0.2 M was performed for 5 min to isolate N_0.3_-ACP. The resulting solid was collected and stored as described above. All the synthetic conditions are collected in Table S1.

### Study of structural stability in water

With the aim to evaluate the structural stability of N_0.3_-ACP, nAp and N_0.3_-nAp, 20 mg of each material were suspended in 20 mL of water (1.0 g L^−1^) and stirred at room temperature during one and three days. At each time, solids were collected by centrifugation (5 min, 4,500 rpm) and freeze-dried without washing. The stability of the materials was evaluated by XRPD and FTIR.

### Calcium release studies

The amount of Ca^2+^ released from the selected nitrogen-doped CaP nanoparticles, namely N_0.3_-ACP and N_0.3_-nAp, and from the undoped material nAp, was monitored by using Ca^2+^-selective electrode methodology (Mettler-Toledo). The operational pH and concentration ranges are 2–12 and 10^–6^–1 M, respectively. Nominal selectivity values for Ca^2+^ vs. alkaline ions (K^+^, Na^+^) are 10^4^. Prior to each experiment, a calibration curve of Ca^2+^ was performed using CaCl_2_.2H_2_O (1, 10, 100 and 1,000 ppm of Ca^2+^), which provided 3% relative errors; KCl 4 M was used as ionic strength adjustment buffer (ISAB). In a typical experiment, 50 mg of CaP nanoparticles were suspended in 50 mL of water to which 2 mL of ISAB were added. The suspension was kept under stirring at room temperature and covered to avoid evaporation. The Ca^2+^ concentration vs. time was monitored in 15 min steps, providing several hundreds of points within days. N_0.3_-ACP released 39.2 mg of calcium per g of material (61.9 mg g^−1^ of PO_4_^3−^) after 60 h of suspension, reaching 10.1% of dissolution [calculated on a Ca_3_(PO_4_)_2_ basis]; in the same time, nAp released 21.1 mg g^−1^ of calcium (30.0 mg g^−1^ of phosphate) and N_0.3_-nAp released 21.9 mg g^−1^ of calcium (31.1 mg g^−1^ of phosphate), reaching 5.5 and 5.6% of dissolution, respectively [calculated on a Ca_5_(PO_4_)_3_(OH) basis]. During the Ca^2+^ release tests, undersaturation conditions, with respect to both ACP and carbonated apatite, were fully ensured (see SI).

### Nitrate release studies

The amount of NO_3_^*–*^ released from the selected nitrogen-doped CaP nanoparticles, namely N_0.3_-ACP and N_0.3_-nAp was monitored by using NO_3_^*–*^-selective electrode methodology (Metler-Toledo). The operational pH and concentration ranges are 2–12 and 10^–5^–1 M, respectively. Nominal selectivity values for NO_3_^*–*^ vs. oxyanions (H_2_PO_4_^*–*^, HPO_4_^2*–*^ and HCO_3_^*–*^) are well above 10^3^. Prior to each experiment, a calibration curve of NO_3_^*–*^ was performed using KNO_3_ as salt (1, 10, 100 and 1,000 ppm of NO_3_^*–*^), which provided less than 5% relative errors; (NH_4_)_2_(SO_4_) 2 M was used as ionic strength adjustment buffer (ISAB). In a typical experiment, 50 mg of CaP nanoparticles were suspended in 50 mL of water to which 2 mL of ISAB were added. The suspension was kept under stirring at room temperature and covered to avoid evaporation. The NO_3_^*–*^ concentration vs. time was monitored in 5 min steps, providing several hundreds of points within days. N_0.3_-ACP delivered 19.5 mg NO_3_^*–*^ per gram of material within the first 10 h of stirring, reaching the 94.2% of the total nitrate amount in the material. N_0.3_-nAp released 2.4 mg NO_3_^*–*^ per gram of material within the first 60 h of stirring (54.6% of total nitrate) and 2.6 mg g^−1^ after 120 h of suspension (58.9% of total nitrate).

### Instrumental characterization

#### Fourier-transform infrared spectroscopy (FTIR)

The infrared spectroscopy data were collected with a Bruker Tensor 25 FTIR spectrophotometer. Elemental (C, H, N) analyses were obtained by a Thermo Scientific Flash 2000 Organic Elemental Analyzer equipped with a microbalance (XP6, Mettler Toledo, at the Centre of Scientific Instrumentation, University of Granada).

#### X-ray powder diffraction (XRPD)

XRPD measurements were performed on samples analysed for structural stability and nutrient release. XRPD data were collected on a Rigaku Miniflex diffractometer using Cu Kα radiation (λ = 1.5418 Å), from 5° to 55° (2θ) with a scan rate of 0.2° min^−1^ and ∆2θ = 0.02°.

#### Transmission electron microscopy (TEM)

TEM images were collected with a LIBRA 120 PLUS (Carl Zeiss SMT) operating at 120 kV. Synthesized nanoparticles collected by centrifugation were ultrasonically dispersed in ethanol and then few drops of the slurry were deposited on 200 mesh copper grids covered with thin amorphous carbon films.

#### Inductively coupled plasma optical emission spectrometry (ICP-OES)

The chemical composition of powdered samples (Ca, P and K) was analysed by ICP-OES (Perkin Elmer OPTIMA 8300). 20 mg of the powdered sample were dissolved in 2 ml of ultrapure nitric acid and then diluted to 100 mL with Milli-Q water. The emission wavelengths were 317.93 nm (Ca), 213.62 nm (P) and 766.49 nm (K).

### Small-angle X-ray scattering measurements (SAXS)

The SAXS measurements were performed at the in-house SAXS instrument at Aarhus University^78^. It uses a rotating Cu anode source, side-by-side Montel multilayer mirrors for monochromatizing and focusing the beam, and a Vantec 500 (Bruker AXS) detector. The collimation consists of two-pinholes, where the one close to the sample is a scatterless pinhole with edges of Ge crystal^79,80^. Thin layers of the powdered samples were picked up by matte acetate Scotch tape and mounted in the beam in the integrated vacuum of the SAXS instrument. A piece of the same tape was measured and subtracted as background. The data treatment was done using the in-house developed SUPERSAXS program package (C.L.P. Oliveira and J.S. Pedersen, unpublished). Details on the modelling and data analysis can be found in the Supplementary Methods.

### Synchrotron WAXTS measurements

Dry powder samples were loaded in glass capillaries (diameter of 0.5 mm) and measured at the X04SA-MS beamline of the Swiss Light Source (Paul Scherrer Institut, Villigen, CH)^81^. WAXTS data were collected in transmission mode in the 2–120 2θ range using a single-photon counting silicon microstrip MYTHEN II detector. The beam energy was set at 16 keV and the operational wavelength (λ = 0.775108 Å) was determined by measuring a silicon powder standard sample (NIST 640c). Separate air and empty capillary scattering measurements were acquired, and the transmission coefficients of the samples experimentally determined (by measuring direct and transmitted beam) whereas that of the glass capillary calculated from the certified composition. Raw data were then corrected for systematic errors and absorption effects; the extra-sample contributions to the diffraction pattern, namely the capillary and the sample environment, were subtracted. After this reduction procedure, data only account for the sample contribution to the diffraction pattern, which is analysed through a total scattering approach based on the Debye scattering equation (DSE).

### The DSE method

The total scattering method here applied relies on the implementation of the Debye scattering equation^52,57^, enabling the computation of diffraction patterns of randomly oriented nanoparticles from the distribution of interatomic distances within the sample, without any assumption on the structural order. The model scattering pattern is calculated as follows:

$$I\left(Q\right)={\sum }_{j=1}^{N}{f}_{j}{(Q)}^{2}{{o}_{j}}^{2}+2{\sum }_{j>i=1}^{N}{f}_{j}(Q){f}_{i}(Q){T}_{j}(Q){T}_{i}(Q){o}_{j}{o}_{i}\frac{sin ({Qd}_{ij})}{{(Qd}_{ij})}$$

where *Q* = 4πsin*θ*/*λ* is the scattering vector amplitude, *θ *is half of the scattering angle 2*θ*, *λ* is the radiation wavelength, *f*_*j*_ is the atomic form factor of atom *j*, *d*_*ij*_ is the interatomic distance between *i* and *j* atom pairs and *N* is the number of atoms in the nanoparticle. *T* and *o* parameters refer to the atomic thermal vibration and site-occupancy, respectively. The first summation accounts for the contribution of the zero distances of each atom from itself, whereas the second summation accounts for the non-zero distances between pairs of distinct atoms. The DSE modelling of nanomaterials is carried out using the DebUsSy Suite^57^ through subsequent steps, following a bottom-up approach. Pseudo-multiplicities *vs* equi-spaced pair distances are encoded in databases, by applying a Gaussian sampling of interatomic distances, according to the algorithm implemented in the Suite for speeding up calculation^82^. At first, for each sample, the hydroxyapatite cell parameters (*a* = *b* ≠ *c*) were determined through the Rietveld refinement method. The hydroxyapatite unit cell, with adjusted cell parameters, was then used as a building block to generate populations of atomistic models of nanocrystals of increasing size. Details of DSE modelling for SAXS and WAXTS analysis can be found in the Supplementary Methods.

## Supplementary information

Supplementary Information

## Data Availability

The datasets generated during and/or analysed during the current study are available from the corresponding author on reasonable request.

## Code availability

The *DebUsSy* program suite is freely available at https://debussy.sourceforge.net.
