# Supplementary material for: The role of nanoparticle structure and morphology in the dissolution kinetics and nutrient release of nitrate-doped calcium phosphate nanofertilizers
Source: Sci Rep. 2020 Jul 24;10:12396. doi: 10.1038/s41598-020-69279-2 (PMC7382453; doi:10.1038/s41598-020-69279-2)
Supplement: Supplementary file 1 — Supplementary Information [file 41598_2020_69279_MOESM1_ESM.pdf]

Supplementary Information

The role of nanoparticle structure and morphology in the dissolution kinetics and nutrient release of nitrate-doped calcium phosphate nanofertilizers

Francisco J. Carmona, Gregorio Dal Sasso, Federica Bertolotti, Gloria B. Ramírez-Rodríguez, José Manuel Delgado López, Jan Skov Pedersen, Norberto Masciocchi* & Antonietta Guagliardi *

*To whom correspondence should be addressed: antonella.guagliardi@ic.cnr.it

Supplementary Methods

SAXS data analysis

SAXS and WAXTS data analysis by the Debye scattering equation (DSE) method

Lattice strain model

Saturation Index

Supplementary Figures

Figures S1-S5

Supplementary Tables

Tables S1-S4

SAXS data analysis

The scattered intensity is given as a function of the modulus of the scattering vector:

$$Q = \frac{4\pi}{\lambda}\sin\theta$$

where λ is the wavelength of the incident radiation and 2θ is the scattering angle. Conventional SAXS analysis was done as a first step. The intensity is expressed as

$$I(Q) = \langle P(Q)\rangle S(Q)$$

where the form factor $\langle P(Q)\rangle$ is related to the average size and the shape of the particles, and the structure factor $S(q)$ is related to concentration and/or aggregation effects.[1] The form factor was taken as that of circular thin discs with a radius R and a thickness T, which for monodisperse and particles with large anisotropy can be written as:

$$P(Q) = \frac{2}{(QR)^2}(1 - J_1(2QR))\left|\frac{\sin(QT/2)}{QT/2}\right|^2$$

where $J_1(\mathrm{x})$ is the Bessel function of first kind and first order. Polydispersity of the thickness T was included using a Zimm-Schulz distribution, which allows an analytical result to be obtained: [2]

$$\langle P(Q)\rangle = \frac{2}{(QR)^2}\left(1 - J_1(2QR)\right)\left(\frac{2\alpha^2}{Q^2(1+Z)(2+Z)}\right)\left(1 - \frac{\cos((1+Z)\,\mathrm{Atan}(Q/\alpha)))}{(1+Q^2/\alpha^2)^{(1+Z)/2}}\right)$$

with $Z = 1/\sigma^2 - 1$, where σ is the relative standard deviation of the distribution and $\alpha = (Z+1)/T$ and T is now the number average thickness. In order to get good fits at the low Q values, concentration effects had to be considered and this was done using a random-phase approximation structure factor: [3]

$$S(q) = \frac{1}{1 + \nu\langle P(Q)\rangle}$$

where ν is an increasing function of the concentration. The model could describe the SAXS data for the nAp and $N_{0.2}$-nAp samples satisfactory, however, the $N_{0.3}$-nAp sample could not be described by this model and it was necessary to include an additional population of thinner platelets jointly to a thicker one, resulting in a bimodal distribution of platelets.

The ACP and $N_{0.3}$-ACP samples have a SAXS intensity with a Q dependence, which is quite different from that of the nAp, $N_{0.2}$-nAp, and $N_{0.3}$-nAp, with a much steeper Q dependence, and a different model had to be used. A model for polydisperse spheres was able to fit the data at high Q, however, due to aggregation and clustering, an additional empirical cluster structure factor was used:

$$S_{cluster}(Q) = S_{HS}(Q, R_{HS}, \eta_{HS}) + A/(1 + (Q\xi)^2)^2$$

Here $S_{HS}(Q, R_{HS}, \eta_{HS})$ is the hard-sphere structure factor[4] that depends on an effective hard-sphere interaction radius R_{HS} and an effective hard-sphere volume fraction η_{HS}, and describes correlations of the particles within the cluster. The last term describes the overall cluster and is taken as the Debye-Bueche expression[5] with a scale factor A in front. The parameter ξ is related to the overall size of the clusters.

Optimization of the scales, a background, and the structural parameters in the models was done using least-squares methods and the error bars on the parameters were obtained as in ref. 1. Average sizes and size dispersions and other parameters or quantities derived by these models are given in Table S3.

SAXS and WAXTS data analysis by the Debye scattering equation (DSE) method

The DSE modelling of CaP nanomaterials here investigated was carried out using the DebUsSy Suite through subsequent steps, following a bottom-up approach. At first, for each sample, the hydroxyapatite cell parameters (*a* = *b* and *c*) were determined through the Rietveld refinement method implemented in the MAUD software,[6] using a spherical harmonics model[7] to phenomenologically describe the finite-size broadening of diffraction peaks. The hydroxyapatite unit cell, with adjusted cell parameters, was then used as a building block to generate populations of atomistic models of nanocrystals of increasing size. Nanocrystals were modelled according to a prismatic shape considering two perpendicular (independent) growth directions, one along the *c* axis and one in the *ab* plane. By varying the *b*/*a* ratio from 1 to 8, the nanocrystals shape can be modelled as an elongated prism (when *a* = *b*), resembling the hexagonal morphology proper of the hydroxyapatite structure, or as a platelet, progressively wider by increasing the *b*/*a* ratio, breaking the hexagonal crystal symmetry as observed in bone or biomimetic apatite samples. For each nanocrystal, sampled interatomic distances were computed and stored in a database used to simulate the diffraction pattern through the Debye equation. A sampling algorithm of the true distances is adopted, thus reducing by order of magnitude the number of terms in the Debye equation without losing accuracy in the calculated pattern; this makes the analysis through the Debye equation affordable in terms of computational time. The simulated scattering patterns were calculated (within a non-interfering particle model approximation) in the SAXS and WAXTS regions using the same (morphological) databases, then compared to the experimental ones. The difference between measured and calculated patterns were minimized by refining a number of adjustable structural and microstructural parameters in the model using the Simplex algorithm.

In particular, platelets with optimized *b*/*a* ratio equal to 7 were used to model SAXS data of nAp, $N_{0.2}$-nAp and $N_{0.3}$-nAp. The lowest Q region (more influenced by aggregation effects) was removed from the DSE fitting (Fig. S4). The nanoplates size distribution was described by a bivariate lognormal function, for which the average size and associated standard deviation for the two independent growth directions (D_{ab}, equivalent diameter of the area in the *ab* plane, and length along the *c* axis) were refined. The thickness of the platelets is retrieved from the D_{ab} values, at known *b*/*a* ratio. Due to the limited content of information of SAXS data, the model of platelets with adjustable width, length and their dispersions is beyond the capability of SAXS modelling. However, extensive tests (presented elsewhere jointly to many additional technical details, which are out of the scope of this work[8]) suggested that the thickness remains a reliable estimate (as also demonstrated by the matching with the conventional analysis) and that SAXS analysis may benefit from reasonable restrains taken from WAXTS analysis.

For the WAXTS model, platelets with optimized *b*/*a* ratio equal to 6 (nAp) or 5 ($N_{0.2}$-nAp, $N_{0.3}$-nAp) were used. The nanocrystals size distribution was described by a bivariate lognormal function, for which the average size and standard deviation in the *ab* plane and the pairs associated to the nanocrystal length along the *c* axis were relaxed. Average sizes and size dispersions and other parameters or quantities derived by the WAXTS-DSE modelling are given in Table 1 of the main text (number-based values) and Table S4 (mass-based values). The site occupancy factors of the two Ca atoms and the isotropic Debye-Waller factor of all atoms were also refined (Table S4). For the WAXTS analysis, the atomistic models of platelets were suitably managed to obtain additional peak broadening due to anisotropic lattice strain, as detailed in the next paragraph.[8]

Lattice strain model

An isotropic lattice strain was modelled through a phenomenological approach,[9] by convoluting the DSE sample pattern with a Gaussian function of width $4\varepsilon \tan\theta$, where $\varepsilon = \Delta r/r$ is the adimensional (adjustable) parameter measuring the radial expansion/contraction of r around its average value. This model did not provide satisfactory matching with the experimental data. An anisotropic strain model was introduced at the atomistic level, as much

as described in ref. [10] Two different (elastic) strain tensors were considered: (1) a strain tensor accounting for two independent components, $\varepsilon_a=\Delta a/a$ (ε_{11} in matrix notation) and $\varepsilon_c=\Delta c/c$ (ε_{33}), along the a and c lattice parameters, in line with the hexagonal crystal symmetry of apatite, for which $\varepsilon_b = \varepsilon_a$ and all the off-diagonal terms of the strain tensor vanishing; [11] (2) a strain tensor accounting for three independent components, $\varepsilon_a=\Delta a/a$ (ε_{11}), $\varepsilon_b=\Delta b/b$ (ε_{22}) and $\varepsilon_c=\Delta c/c$ (ε_{33}), along a, b and c lattice parameters, in line with the morphological symmetry of platelets (breaking the structural one). Accordingly, an additional (not vanishing) off-diagonal term of the strain tensor ($\varepsilon_{13} = \varepsilon_{31}$) would have been considered but it was neglected being typically smaller than the diagonal terms. The lattice parameters were assumed (in both models) to be distributed about their average values according to a Gaussian function of width Δx, properly binned in order to avoid undesired effects on the DSE simulation, due to a coarse binning; limiting the computational time was also an important issue, leading to the choice of 30 bins as the best compromise. Accordingly, for each D_{ab}, L_c nanocrystal size combination of the bivariate population, 30 atomistic models were managed and combined into a single set of pseudo-multiplicities *vs* equi-spaced pair distances, according to the algorithm implemented in the Debussy Suite for speeding up calculation by applying a Gaussian sampling of interatomic distances. A grid search algorithm exploring the GOF statistical value (matching the agreement between simulated and experimental patterns) while varying the strain parameters (with a 0.05% sampling step) was applied to find the best strain model. The model 2, accounting for doping-induced effects driven by both structural and morphological influences, provided the best agreement; the ε_a, ε_b and ε_c values are reported in Table S4 for all nanocomposites.

Saturation index

Whether, during our dissolution experiments, saturation conditions are approached, or reached, it was calculated by comparing the actual (measured) [Ca2+] concentration, with the maximum solubility calculated at equilibrium. Taking the apatite formulation as $Ca_5(PO_4)_3(OH)$, and the thermodynamic constants defined below, it is possible to demonstrate that in the 5 < pH < 8 range, the maximum concentration of calcium obtained by apatite dissolution in saturated conditions is:

$$[Ca^{2+}] = (\frac{125}{27}[H^+]^7 K_{sp}/\,[K_w\,(K_{a2}K_{a3})^3])^{1/8}$$

where K_w = 1.01 10^{-14} (ionic product of water), K_{a2} = 6.2 10^{-8} and K_{a3} = 3.6 10^{-13} (second and third acid dissociation constants of H_3PO_4 at 25°C) where taken from ref. 12. The solubility product of apatites, K_{sp} = $[Ca^{2+}]^5[PO_4^{3-}]^3[OH^-]$ indicates that the equilibrium value of the $[Ca^{2+}]$ concentration is heavily pH-dependent, both in the phosphate and hydroxyl terms, and, due to partial calcium complexation by $[CO_3^{2-}]$ or $[HCO_3^-]$ in solutions generated by suspending carbonated apatites in water, also in carbonate content. According to ref.13, the K_{sp} values of carbonated apatites strongly depend on carbonate content. By slightly rearranging the originally one, the following equation was herein used to calculate the pK_{sp} for our samples:

$$pK_{sp} = ½\cdot\{118.65 - [44/60 \cdot (CO_3^{2-}\,w\%)^{2.4176}\}$$

Specifically, for CO_3^{2-} = 7.4 w% (Table S2) and pH = 7.1, the equilibrium value $[Ca^{2+}]_{eq}$ = 85.0 mg l^{-1} is calculated.

The experimentally determined values for our nanocomposites are $[Ca^{2+}]$ < 22 mg l^{-1}, at pH = 7.1; the Saturation Index $S = (IP/K_{sp})^{1/9}$ < 0.30 was calculated (according to ref. 14), where IP is the Ionic Product and K_{sp} the Solubility Product. Similarly, the Relative Saturation Index: $\sigma = S-1$ = –0.70, or less, was obtained. Both values indicate that, in our dissolution process, saturation is never reached.

Supplementary Figures

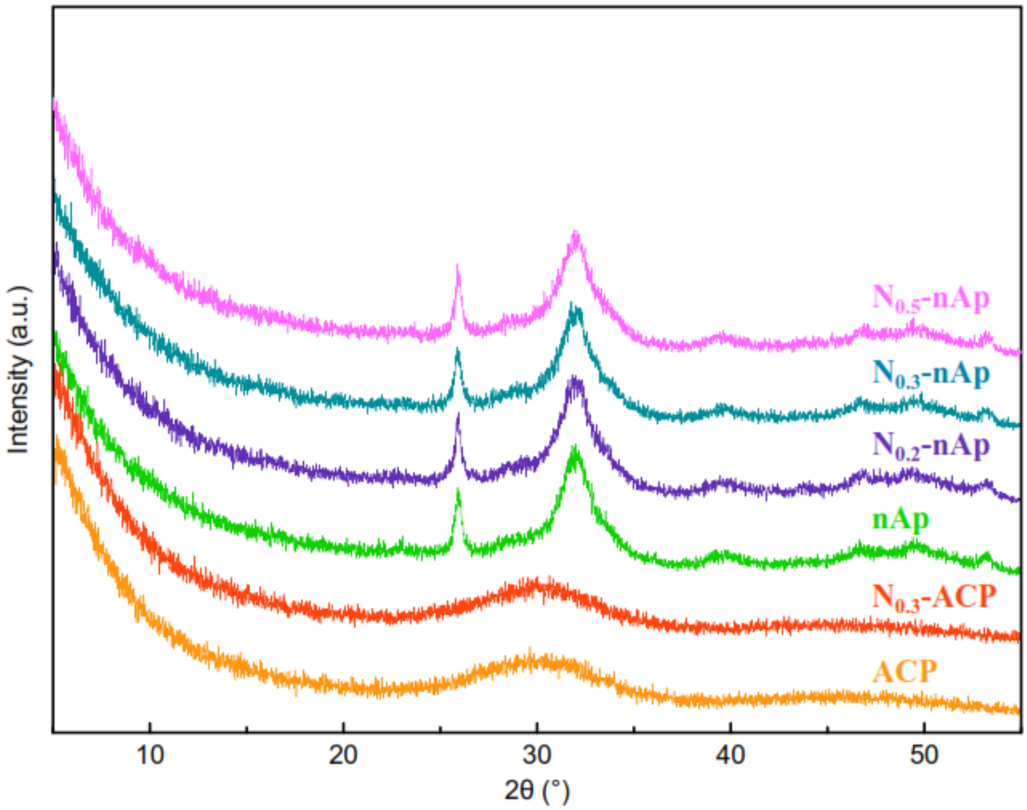

Fig. S1. Lab X-ray powder diffractograms of biomimetic CaP nanoparticles. The patterns of ACP and $N_{0.3}$-ACP (yellow and orange curves, respectively) confirm the amorphous nature of the materials. On the other hand, XRPD data of nAp and N_x-nAp materials show the typical diffraction pattern of nanosized hydroxyapatite. The absence of reflections due to other crystalline phases confirms the purity of all materials.

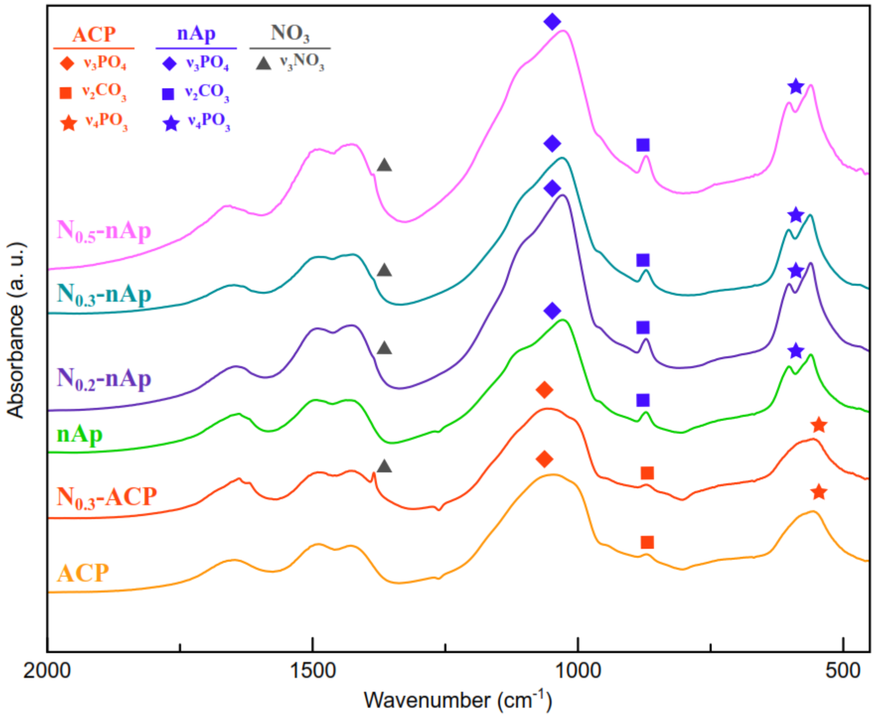

Fig. S2. FTIR spectra of CaP nanoparticles. Both ACP and $N_{0.3}$-ACP materials (orange and red curves, respectively) show a strong absorption band covering the 1200–900 cm^{-1} range (associated to $\nu_3(PO_4)$, red diamonds) and the $\nu_4(PO_4)$ bending band (630–500 cm^{-1}, red stars), which are typical of amorphous calcium phosphate.[15] Additional bands, associated to CO_3^{2-} vibrational modes (~ 1485 cm^{-1} and ~ 1425 cm^{-1} [$\nu_3(CO_3)$] and at 868 cm^{-1} [$\nu_2(CO_3)$] (red squares))[16] suggest the presence of carbonate ions admixed with the ACP material. On the other hand, the analysis of nanocomposite materials (green, purple, turquoise and pink curves) shows several PO_4^{3-} bands typical of apatite: strong absorption bands in the 1100 to 1000 cm^{-1} region, associated to the antisymmetric $\nu_3(PO_4)$ stretching (blue diamonds), and in the 650 to 550 cm^{-1} one, for antisymmetric $\nu_4(PO_4)$ bending (blue stars).[15] Additional bands, associated to CO_3^{2-} vibrational modes,[17] can be identified at ~ 1485 cm^{-1} and ~ 1425 cm^{-1} [$\nu_3(CO_3)$] and at ~ 871 cm^{-1} [$\nu_2(CO_3)$] (blue squares), indicating the partial substitution of PO_4^{3-} by carbonate ions in the apatite crystal lattice, with very minor OH^- groups replacement.[18] These results are well in agreement with the experimental observations showing that carbonate is the major substituent in biological apatite and confirm that biomimetic CaP are obtained.[19] Additionally, the presence of nitrate ions in the isolated nitrate-doped nanomaterials, namely $N_{0.3}$-ACP and N_x-nAp, is confirmed by the presence of the sharp (though weak) IR band associated to the ν_3NO_3 asymmetric stretching falling at 1384 cm^{-1} (grey triangles).[20]

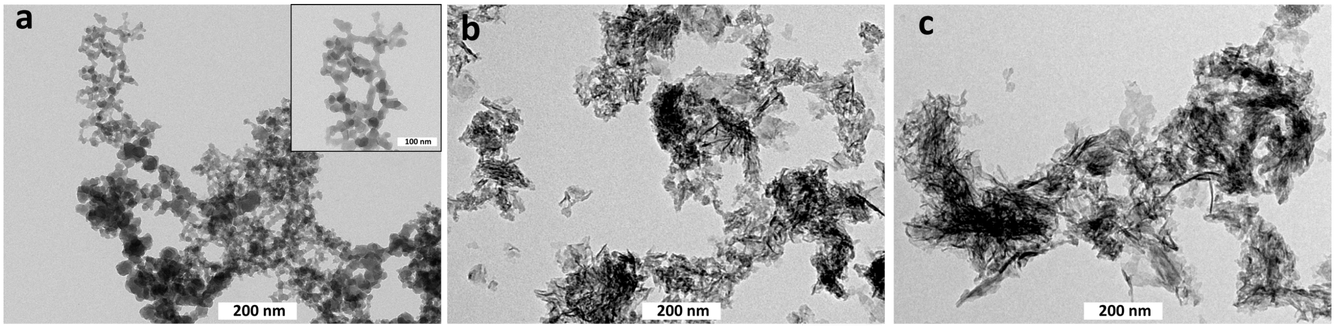

Fig. S3. TEM images of dry samples showing rounded nanoparticles of ACP (a) and nanoplates of nAp (b) and $N_{0.3}$-nAp (c).

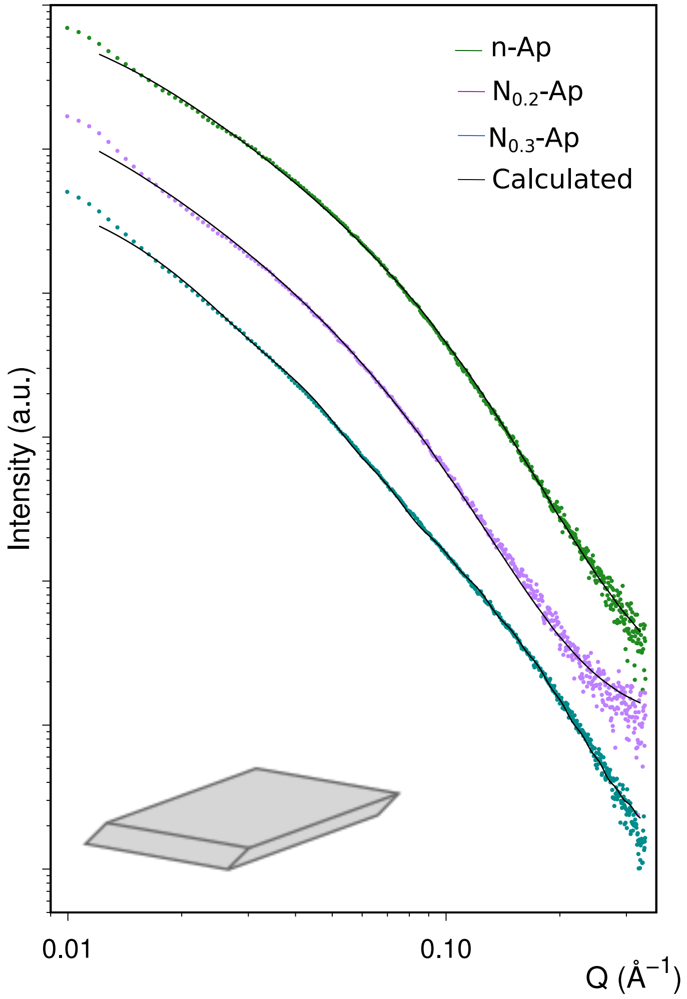

Fig. S4. SAXS data (log-log scale) of nAp, $N_{0.2}$-nAp and $N_{0.3}$-nAp samples and DSE modeling based on atomistic models of nanoplates. Number- and mass-based average thickness (*T*), width (*W*) and length (*L*) (in nm), and relative dispersions (σ_T/T, σ_W/W and σ_L/L) are reported in Table 1 of the main text and Table S4.

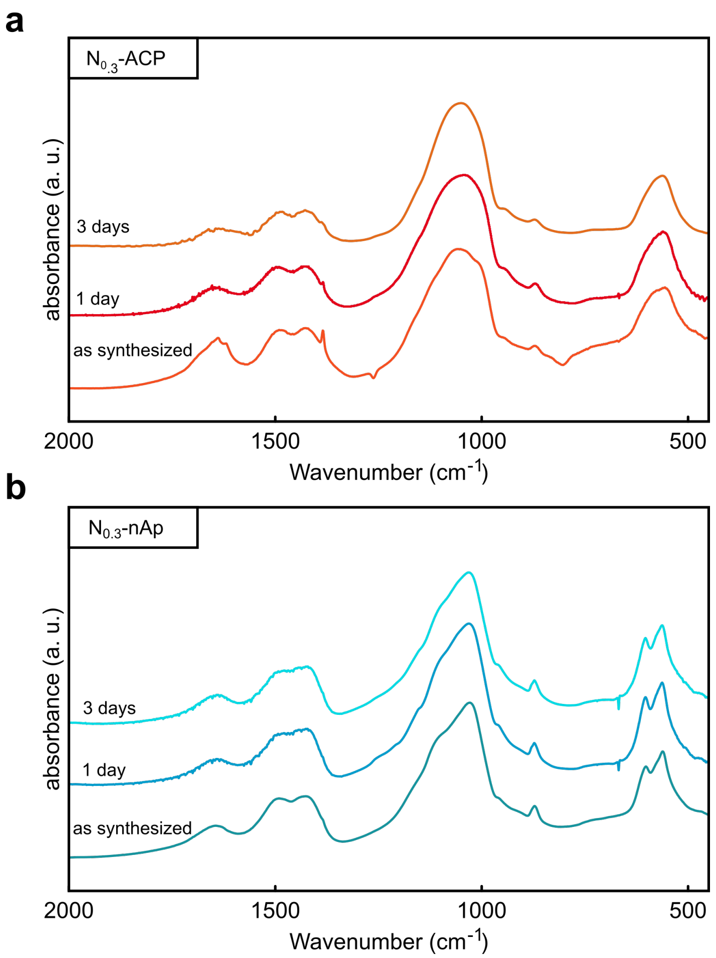

Fig. S5. FTIR spectra of a) $N_{0.3}$-ACP and b) $N_{0.3}$-nAp measured as synthesized, after 1 and 3 days of suspension in water at room temperature. The FTIR spectra of both materials after 3 days of suspensions are similar than their pristine ones, respectively, certifying the chemical stability of both systems.

Supplementary Tables

Table S1. Synthetic conditions for the undoped and N_x-doped calcium phosphate samples. ACP and N_x- ACP labels indicate fully amorphous samples; nAp and N_x-nAp labels refer to samples containing crystalline apatite.

Sample	Solution A		Solution B			Maturation time (min)
	$CaCl_2$	$Ca(NO_3)_2$	K_2HPO_4	Na_2CO_3	KNO_3	
ACP	0.2		0.12	0.1	-	5
$N_{0.3}$-ACP	-	0.2	0.12	0.1	0.2	5
nAp	0.2	-	0.12	0.1	-	1440
$N_{0.2}$-nAp	-	0.2	0.12	0.1	-	1440
$N_{0.3}$-nAp	-	0.2	0.12	0.1	0.4	1440
$N_{0.5}$-nAp	-	0.2	0.12	0.1	0.6	1440

Table S2. Nitrate, carbonate, calcium and phosphorus content in biomimetic nanoparticles.

Sample	NO_3^- (wt %)[a]	CO_3^{2-} (wt %)[a]	Ca (wt%)[b]	P (wt%)[b]	Ca/P[b]	K (wt%)[b]	Na (wt%)[b]
ACP	-	-	21.37 (0.16)	14.48 (0.22)	1.57	0.80 (0.06)	0.97 (0.15)
$N_{0.3}$-ACP	2.12	7.44	28.28 (0.42)	13.51 (0.13)	1.62	1.53 (0.08)	0.98 (0.20)
nAp	0.0	7.40	29.93 (0.54)	14.41 (0.18)	1.64	0.25 (0.15)	0.31 (0.09)
$N_{0.2}$-nAp	0.39	8.70	30.26 (0.54)	13.68 (0.13)	1.71	1.01 (0.05)	0.97 (0.26)
$N_{0.3}$-nAp	0.44	7.34	30.43 (0.85)	14.51 (0.41)	1.62	0.21 (0.11)	0.31 (0.10)
$N_{0.5}$-nAp	0.44	7.54	-	-	-	-	-

[a] Calculated by elemental analysis; [b] Calculated by ICP-OES

Table S3. Number-based average thickness (*T*) and diameter (*D*) of N_x-nAp and ACP samples provided by classical SAXS analysis using a finite disk-shape and a spherical model, respectively. The relative thickness dispersion (σ_T/T) of disc was modeled using a Zimm-Schultz distribution function, whereas the disc diameters were assumed to be monodisperse by fixing σ_D/D = 0.1. The ν parameter describes concentration effects. For the hard-sphere model applied to the amorphous (ACP and $N_{0.3}$-ACP) samples, the meaning of the refined parameters is given in the supplementary text.

Sample	Form factor	*T* (nm)	σ_T/T (%)	*D* (nm)	*ν*	χ^2					
nAp	Disc	2.30 (5)	50 (2)	44.0 (1)	2.32(6)	3.08					
$N_{0.2}$-nAp	Disc	2.42 (6)	57 (2)	44.0 (1)	0.49(6)	5.20					
$N_{0.3}$-nAp	Disc	3.8 (9)	58 (15)	44.0 (1)	0.49(6)	9.02					
	Disc	1.3 (3)	35 (33)	44.0 (1)	0.49(6)	-					
					R (nm)	*σ/R* (%)	R_{HS}/R	η_{HS}	*A*	*ζ* (nm)	*R* aggregate (nm)
ACP	Sphere				6.8(1)	63	0.7(1)	0.2(3)	0.8(6)	6.0(2)	19
$N_{0.3}$-ACP	Sphere				6.5(5)	62	0.6(2)	0.0(6)	2.3(4)	7.6(3)	24

Table S4. Mass-based average thickness (*T*), width (*W*) and length (*L*) and relative dispersions ($\sigma_T/T = \sigma_W/W$, σ_L/L) of entire NPLs (from SAXS-DSE) and apatite (Ap) NPLs (from WAXTS-DSE); agreement indices (χ^2 and Goodness of Fit, *Gof,* are provided for the best models. Anisotropic lattice strain parameters (ε_a, ε_b, ε_c) derived from the hyperspace exploration described in the Supplementary Methods. Isotropic Debye-Waller factors (*D-W*, Å^2) for Ca_1, Ca_2, P and O atoms and site occupancy factors (*sof*) for Ca_1, Ca_2 from WAXTS analysis. The Ca/P ratio refers to the crystalline fraction of nanocomposites and is calculated from the refined site occupancy factors of Ca ions.

**SAXS**	*T* (nm)	*W* (nm)	$\sigma_T/T = \sigma_W/W$ (%)	*L* (nm)	σ_L/L (%)	χ^2	T_{SAXS} - T_{WAXTS} (nm)	W_{SAXS} - W_{WAXTS} (nm)	L_{SAXS} - L_{WAXTS} (nm)
nAp	3.21	25.97	45.64	17.69	76.21	1.85	0.23	5.30	-0.1
$N_{0.2}$-nAp	3.83	30.96	50.83	19.78	70.82	2.43	0.54	11.12	1.71
$N_{0.3}$-nAp	5.81	46.95	35.84	31.01	11.05	1.09	0.55	16.55	11.13
	1.75	14.14	31.81	39.92	4.12		0.05	4.31	19.44
**WAXTS**									
						Gof	ε_a	ε_b	ε_c
nAp	2.98	20.67	42.44	17.75	73.98	5.25	0.022	0.010	0.0040
$N_{0.2}$-nAp	3.29	18.97	48.70	18.07	69.21	5.21	0.022	0.014	0.0045
$N_{0.3}$-nAp	5.26	30.40	29.97	19.88	69.36	8.24	0.022	0.010	0.0040
	1.70	9.83	2747	19.88	69.36				
D-W Factors (Å^2)	Ca_1	Ca_2	*P*	O_{PO4}	O_{OH}		$Ca_{1\,sof}$	$Ca_{2\,sof}$	*Ca/P*
nAp	0.66	1.44	1.80	0.69	2.87		0.97	1.00	1.65
$N_{0.2}$-nAp	0.53	1.24	1.68	0.56	3.90		0.97	0.98	1.63
$N_{0.3}$-nAp	0.47	1.29	2.50	0.49	4.46		0.97	1.00	1.65
	1.10[a]	1.30	1.24	1.11	1.34		1.00	0.95	1.62

[a] The D-W factors for the smallest nanoplates have been fixed for all atoms.

References

1. Pedersen, J. S., Analysis of Small-Angle Scattering Data from Colloids and Polymer Solutions: Modeling and Least-Squares Fitting. *Adv. Colloid Interface Sci.* **70**, 171–210 (1997).
2. Bünger, M. H., Oxlund, H., Hansen, T. K., Sørensen, S., Bibby, B. M., Thomsen, J. S., Langdahl, B. L., Besenbacher, F., Pedersen J. S. & Birkedal, H., Strontium and Bone Nanostructure in Normal and Ovariectomized Rats Investigated by Scanning Small-Angle X-Ray Scattering. *Calcif. Tissue Int.* **86**, 294–306 (2010).
3. Zimm, B. H., The Scattering of Light and the Radial Distribution Function of High Polymer Solutions. *J. Chem. Phys.*, **16**, 1093–1099 (1948).
4. Kinning, D. J. & E. L. Thomas, Hard-Sphere Interactions between Spherical Domains in Diblock Copolymers. *Macromolecules* **17**, 1712–1718 (1984).
5. Debye, P. & Bueche, A. M., Scattering by an Inhomogeneous Solid. *J. Appl. Phys.* **20**, 518–525 (1949).
6. Lutterotti, L., Total Pattern Fitting for the Combined Size–Strain–Stress–Texture Determination in Thin Film Diffraction. *Nucl Instrum Methods Phys Res B* **268**, 334–340 (2010).
7. Popa, N. C., The (hkl) Dependence of Diffraction-Line Broadening Caused by Strain and Size for All Laue Groups in Rietveld Refinement. *J. Appl. Crystallogr.* **31**, 176–180 (1998).
8. Bertolotti, F., Carmona, F. J., Dal Sasso, G., Ramírez-Rodríguez, G. B., Delgado López, J. M., Pedersen, J. S., Ferro, F., Masciocchi, N. & Guagliardi, A., On the amorphous layer in bone mineral and biomimetic apatite: A combined small- and wide-angle X-ray scattering analysis*,* *Acta Biomater.* (2020), doi:10.1016/j.actbio.2020.04.026.
9. Tsybulya, S. V., Cherepanova S. V. & Kryukova, G. N., Full Profile Analysis of X-Ray Diffraction Patterns for Investigation of Nanocrystalline Systems. In *Diffraction Analysis of the Microstructure of Materials*; Mittemeijer, E. J., Scardi, P., Eds.; Springer: New York, pp 93–123 (2004).
10. Ulbrich, K. F., Bertolotti, F., Masciocchi, N., Cervellino, A., Guagliardi, A. & Campos, C. E. M., A Comprehensive Structural and Microstructural Investigation of a New Iron–Telluride Nano Phase. *J. Mater. Chem. C* **6**, 3047–3057 (2018).
11. Nye, J. F., Physical Properties of Crystals: Their Representation by Tensors and Matrices; Oxford University Press: New York (1985)**.**
12. Whittern, K. W., Davis, R. E., Peck, M. L. & Stanley, G. G., *General Chemistry 7th Edition; Thomson Learning, Inc.* (2004).
13. Ito, A., Maekawa, K., Tsutsumi, S., Ikazaki, F. & Tateishi, T., *J. Biomed. Mater. Res*. **36**, 522-528 (1997).
14. Tang, R., Wang, L., Orme, C. A., Bonstein, T., Bush, P. J. & Nancollas, G. H., *Angew. Chem. Int. Ed. Engl.*, **43**, 2697-2701 (2004).
15. Stutman, J. M., Termine, J. D. & Posner, A. S., Vibrational Spectra and Structure of Phosphate Ion in Some Calcium Phosphates. *Trans. N. Y. Acad. Sci.* **27**, 669–675 (1965).
16. C. Rey, C. Combes, C. Drouet and D. Grossin, Bioactive Ceramics: Physical Chemistry; In *Comprehensive Biomaterials*; Ducheyne, P., Healy, K., Hutmacher, D., Grainger, D. E., Kirkpatrick, J., Eds.; Elsevier Ltd., 2011.
17. Rey, C., Collins, B., Goehl, T., Dickson, I. R. & Glimcher, M. J., The Carbonate Environment in Bone Mineral: A Resolution-Enhanced Fourier Transform Infrared Spectroscopy Study. *Calcif. Tissue Int. 45*, 157–164 (1989)
18. Antonakos, A., Liarokapis, E. & Leventouri, T., Micro-Raman and FTIR Studies of Synthetic and Natural Apatites. *Biomaterials* **28**, 3043–3054 (2007).
19. Dorozhkin, S. V. & Epple, M., Biological and Medical Significance of Calcilum Phosphates. *Angew. Chem. Int. Ed.* **41**, 3130–3146 (2002).

20. Colthup, N. B., Daly, L. H. & Wiberley, S. E., *Introduction to Infrared and Raman Spectroscopy*; Academic Press Inc.: Burlington (2012).
